# Efficacy of bacteriophage treatment against carbapenem-resistant *Acinetobacter baumannii* in *Galleria mellonella* larvae and a mouse model of acute pneumonia

**DOI:** 10.1186/s12866-019-1443-5

**Published:** 2019-04-02

**Authors:** Jongsoo Jeon, Jong-Hwan Park, Dongeun Yong

**Affiliations:** 1 Department of Laboratory Medicine and Research Institute of Bacterial Resistance, Y, Seoul, Republic of Korea; 2 Laboratory Animal Medicine, College of Veterinary Medicine, Chonnam National University, Gwang-ju, 61186 Republic of Korea; 3 Department of Laboratory Medicine and Research Institute of Bacterial Resistance, Yonsei University College of Medicine, 50-1Yonsei-ro, Seodaemun-gu, Seoul, 03722 South Korea

**Keywords:** Bacteriophage, Carbapenem, *Acinetobacter baumannii*, *Myoviridae*, Phage therapy, *Galleria mellonella*, Mouse acute pneumonia

## Abstract

**Background:**

*Acinetobacter baumannii* is an opportunistic pathogen that causes serious nosocomial infection in intensive care units. In particular, carbapenem-resistant *A. baumannii* (CRAB) strains have been increasing in the past decade, and they have caused major medical problems worldwide. In this study, a novel *A. baumannii* lytic phage, the YMC 13/03/R2096 ABA BP (phage Βϕ-R2096), which specifically causes the lysis of CRAB strains, was characterized in detail in vitro and in silico, and the in vivo effectiveness of phage therapy was evaluated using *Galleria mellonella* and a mouse model of acute pneumonia.

**Results:**

The *A. baumannii* phage Βϕ-R2096 was isolated from sewage water using CRAB clinical strains selected from patients at a university hospital in South Korea. The complete genome of the phage Βϕ-R2096, which belongs to the *Myoviridae* family, was analyzed. Phage Βϕ-R2096 inhibited bacterial growth in a dose-dependent manner and exhibited high bacteriolytic activity at MOI = 10. In the evaluation of its therapeutic potential against CRAB clinical isolates using two in vivo models, phage Βϕ-R2096 increased the survival rates of both *G. mellonella* larvae (from 0 to 50% at 24 h) and mice (from 30% with MOI = 0.1 to 100% with MOI = 10 for 12 days) in post-infection of CRAB. In particular, phage Βϕ-R2096 strongly ameliorated histologic damage to infected lungs, with bacterial clearance in the lungs observed on day 3 postinfection in the mouse acute pneumonia model. Moreover, in vivo studies revealed no mortality or serious side effects in phage-treated groups.

**Conclusion:**

The results of this study strongly suggest that phage Βϕ-R2096, a novel *A. baumannii* lytic phage, could be an alternative antibacterial agent to control CRAB infections. This study is the first report to compare in vivo evaluations (*G. mellonella* larvae and a mouse acute pneumonia model) of the therapeutic efficacy of a phage against CRAB infections.

**Electronic supplementary material:**

The online version of this article (10.1186/s12866-019-1443-5) contains supplementary material, which is available to authorized users.

## Background

The emergence and rise of antibiotic-resistant bacteria related to the use of broad-spectrum antibiotics has been reported [1–3]. Recently, pandrug-resistant pathogens, which are resistant to all commercially available antibiotics, have become significant therapeutic challenges worldwide [4].

*Acinetobacter baumannii*, a Gram-negative coccobacillus, is an important global nosocomial pathogen species that causes infections such as bacteremia, pneumonia, urinary tract infections, wound infections, and meningitis in critically immunocompromised patients in intensive care units (ICUs) [5]. The rapid spread of multidrug-resistant (MDR) *A. baumannii*, one of the ESKAPE (***E****nterococcus faecium,* ***S****taphylococcus aureus,* ***K****lebsiella pneumoniae,* ***A****cinetobacter baumannii,* ***P****seudomonas aeruginosa*, and ***E****nterobacter* species) pathogens, is of great concern in hospitals around the globe [6, 7].

Carbapenems have been the most effective antibiotics against the serious infections caused by *Acinetobacter* spp.; however, carbapenem resistance rates among *A. baumannii* isolates have increased significantly in many countries, including the USA [8], China [9], and South Korea [10, 11], since the first reported emergence in New York, USA, in 1991 [12]. Infections caused by carbapenem-resistant *A. baumannii* (CRAB) are difficult to treat due to limited of therapeutic options, and they are associated with high mortality and economic costs driven by long hospital stays [13–16].

*Acinetobacter baumannii*’s ability to acquire resistance has increased rapidly. Carbapenem resistance is caused by several mechanisms, including the loss of outer membrane proteins, overexpression of efflux pumps and metallo-beta-lactamase [17, 18]. The carbapenem resistance of *A. baumannii* isolates is mostly due to the production of OXA-type carbapenemases (class D carbapenemase-hydrolyzing oxacillinases) [19–21].

Colistin is an alternative agent that retains high activity against all Gram-negative bacilli, including MDR *A. baumannii* [22]. However, it is limited by dose-limiting toxicity and efficacy. Moreover, since the first report in the Czech Republic in 1999, increasing incidents of *A. baumannii* resistance to colistin have been reported in many countries [23–26]. Therefore, the need to develop novel antibacterial agents and strategies to control hospital infections caused by MDR *A. baumannii* is urgent [27–31].

Bacteriophages (phages) are natural viruses that infect bacteria and exist as one of the most abundant biological entities in the biosphere [32]. Since their discovery by Frederick Twort in 1915 and Felix d’Herelle in 1917, clinical approaches for phage therapy have been reported in the USA, Georgia, Poland and Russia; however, phage therapy declined sharply with the introduction of antibiotics in the 1940s [31, 33]. Currently, phages have been reviewed for application as novel alternative agents to combat antimicrobial pathogen challenges caused by the emergence and increase of antibiotic resistance worldwide [34–36].

Since Soothill et al. [37] first reported that phage BS46 had therapeutic potential in vivo to treat systemic infections caused by *A. baumannii*, researchers have studied lytic phages for MDR *Acinetobacter* spp. and suggested them as alternative therapeutics and environmental disinfectants for hospital ICUs [38–44].

Recently, the emergence of phage-resistant mutants to single phages through the mechanisms of phage-resistance has been one of the major concerns in the phage therapy [45]. For this reason, the application of phage cocktails has been used to significantly reduce the evolution of resistant bacteria, and to maintain higher lytic efficacy [46]. Nevertheless, for the fundamental data to formulate a safe and effective phage cocktail, it is important to isolate new bacteriophages and to accumulate information on the characterization of individual phages from experiments in vitro and in vivo [47].

In the present study, we isolated and characterized a novel *A. baumannii* phage, Βϕ-R2096, in vitro and in silico, including its bacteriolytic activity and a whole genome sequence analysis. We also evaluated the in vivo therapeutic potential of phage Βϕ-R2096 against CRAB infection. A *Galleria mellonella* model has been used for several years as a tool to assess the virulence of bacterial pathogens and evaluate the therapeutic efficacy of phages against bacterial infections [48–51]. Therefore, we performed in vivo evaluations of the phage therapy using both the *Galleria mellonella* model and a mouse model of acute pneumonia. To the best of our knowledge, this study is the first to confirm experimental details comparing the therapeutic effects of an *A. baumannii* phage against CRAB clinical strains using *G. mellonella* (wax worms) and a mouse model of acute pneumonia.

## Results

### Characterization of carbapenem-resistant *Acinetobacter baumannii* clinical isolates

The 20 CRAB clinical isolates were resistant to ceftazidime, cefepime, cefotaxime, imipenem, meropenem, piperacillin-tazobactam, and cotrimoxazole, but not colistin and tigecycline. Specifically, the host bacterium of phage Bϕ-R2096, *A. baumannii* YMC13/03/R2096, was resistant to piperacillin-tazobactam, ceftazidime, cefotaxime, cefepime, imipenem, meropenem, gentamicin, amikacin, levofloxacin, and cotrimoxazole, but not ampicillin-sulbactam, colistin, minocycline, or tigecycline (Additional file 1: Table S1). PFGE of 31 carbapenem-resistant and -susceptible *A. baumannii* strains showed different clonality with distinguishable restriction patterns (Additional file 1: Figure S1), and MLST analysis of the 20 CRAB strains, including *A. baumannii* YMC13/03/R2096, indicated that they were sequence type (ST) 357 (allelic profile 1–12–3-2-2-145-3), which belongs to European clone II. All strains had a *bla*_OXA-66_-like gene, which confers carbapenem resistance (Additional file 1: Table S1). We used *A. baumannii* YMC13/03/R2096 for the in vitro characterization and in vivo study of phageBϕ-R2096.

### Characterization of phage Bϕ-R2096 infecting carbapenem-resistant *A. baumannii* strains

**Fig. 1:**
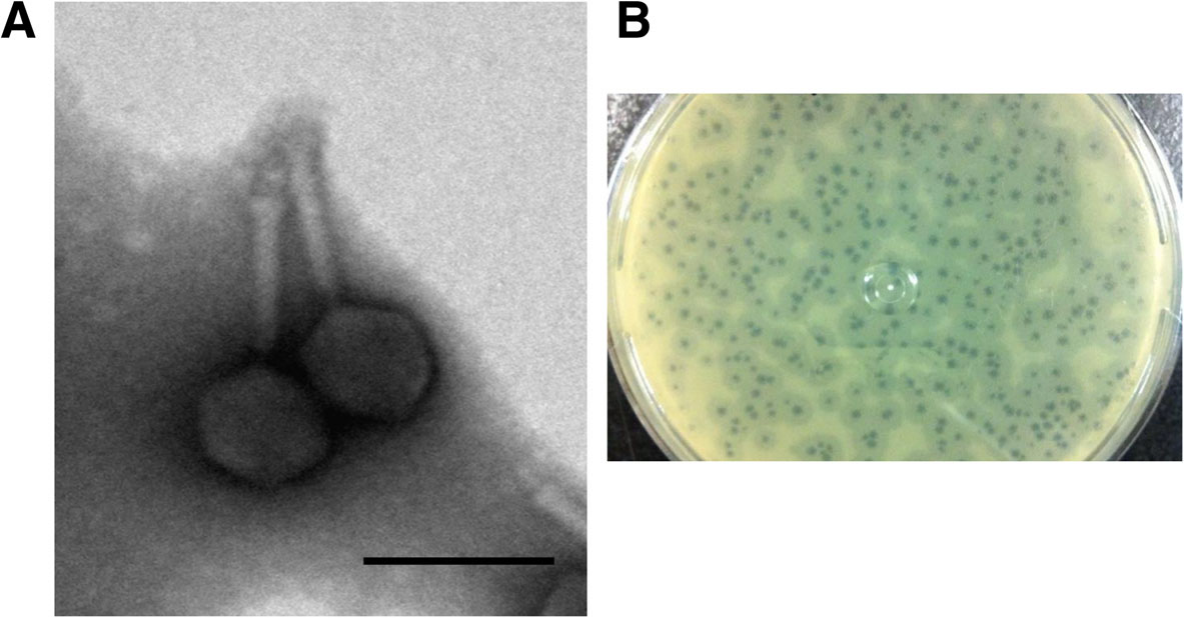
The morphology (**a**) of *A. baumannii* phage Bϕ-R2096 as shown by transmission electron microscopy and plaque formation (**b**) of *A. baumannii* phage Bϕ-R2096. The phage was placed onto a carbon-coated copper grid and negatively stained with 2% uranyl acetate for 15 s. The scale bar = 100 nm

As shown Fig. 1a, electron microscopy indicated that phage Bϕ-R2096 belongs to the *Myoviridae* family, with an isometric head approximately 60 nm in diameter and a contractile tail approximately 89 nm in length (*n* = 15). The phage Bϕ-R2096 formed clear plaques of 2-3 mm, and observed plaque-surrounding halos on agar plates. In the adsorption rate and one step growth curve analysis, phage Bϕ-R2096 exhibited an adsorption rate of 83% within 2 min and 95% within 5 min (Additional file 1: Figure S2 A), a latent period of 50 min, and a burst size of 142 PFU per infected cell (Additional file 1: Figure S2 B). Its temperature and pH stability were evaluated in the 25–70 °C and pH 4–10 ranges (Additional file 1: Figure S3). Phage Bϕ-R2096 showed high stability at 25 °C (> 99%) for up to 9 h and maintained activities of 58, 47, and 24% at 40 °C, 50 °C, and 60 °C, respectively, for up to 9 h, but it showed no activity at 70 °C at any time point (Additional file 1: Figure S3 A). In the pH stability test, > 60% of phage Bϕ-R2096 was retained at pH 7, pH 7.5, and pH 8, and it also showed significant stability (> 40%) at pH 10 during the 10 month study (Additional file 1: Figure S3 B).

### Host spectrum test of *A. baumannii* phage Bϕ-R2096

In the host spectrum assay of phage Bϕ–R2096 against 40 clinical strains (20 carbapenem-resistant *A. baumannii*, 11 carbapenem-susceptible *A. baumannii*, 3 carbapenem- and colistin-resistant *A. baumannii*, 3 carbapenem-resistant *P. aeruginosa*, and 3 carbapenem-resistant *E. coli*), phage Bϕ–R2096 formed a clear zone on 16 of the 20 carbapenem-resistant *A. baumannii* strains and 1 of the 3 carbaepenem- and colistin-resistant *A. baumannii* strains with EOP of ≥0.5 (Table 1). However, we could not measure the EOP in the other 23 isolates. Thus, phage Bϕ-R2096 has strong species and strain specificity and is a relatively broad host-spectrum phage for carbapenem-resistant *A. baumannii* isolates.

**Table 1:** Clinical strains used in this study and their sensitivity to *A. baumannii* phage Bϕ-R2096

Species and strains		Sensitivity^a^to phage Bϕ-R2096	EOP^b^	Species and strains		Sensitivity to phage Bϕ-R2096	EOP
Carbapenem-resistant *A. baumannii*	YMC13/03/R2096	++	1.0	Carbapenem-susceptible *A. baumannii*	YMC13/05/R728	–	
	YMC13/01/R1400	++	0.9		YMC13/05/R550	–	
	YMC13/01/R1224	+	0.6		YMC13/01/R588	–	
	YMC13/01/R1919	+	0.6		YMC13/06/R2026	–	
	YMC13/01/R187	+	0.5		YMC13/01/R722	–	
	YMC13/01/R2058	+	0.6		YMC13/06/R1660	–	
	YMC13/01/R1238	+	0.7		YMC13/04/R3097	–	
	YMC13/01/R249	++	0.9		YMC13/01/R3428	–	
	YMC13/01/R280	++	0.9		YMC13/05/R407	–	
	YMC13/01/R224	++	0.8		YMC13/04/R3148	–	
	YMC13/01/R656	+	0.6		YMC13/01/R3291	–	
	YMC13/03/R12096	+	0.7	Colistin-resistant *A. baumannii*	YMC13/07/ R3044	+	0.5
	YMC13/01/R317	+	0.5		YMC13/08/R2633	–	
	YMC13/01/R129	+	0.5		YMC13/09/R888	–	
	YMC13/01/R3197	+	0.6	Carbapenem-resistant *Pseudomonas aeruginosa*	YMC13/01/B10214	–	
	YMC13/04/B720	+	0.6		YMC13/01/ B11605	–	
	YMC13/02/R291	–			YMC13/03/ B9708	–	
	YMC13/02/R319	–		Carbapenem-resistant *Escherichia coli*	YMC13/01/ B9566	–	
	YMC13/02/R401	–			YMC13/01/ B10710	–	
	YMC13/02/R427	–			YMC13/01/ B11097	–	

^a^Phage activity against collected bacteria: ++, clear plaque; +, turbid plaque; −, no plaque

^b^The efficiency of plating (EOP) was calculated as the titer (PFU/ml) on the test bacteria strain divided by titer (PFU/ml) on host bacteria strain

### Host cell lytic activity test of phage Bϕ–R2096

**Fig. 2:**
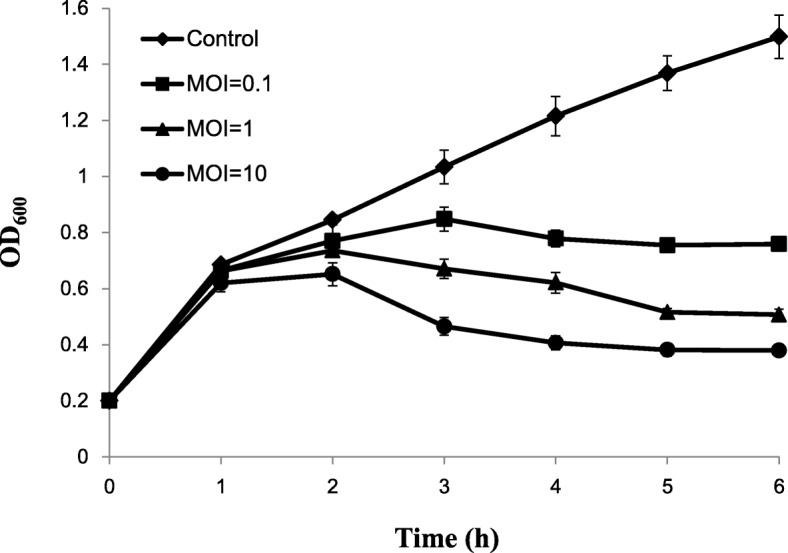
Time course of cell lysis by *A. baumannii* phage Bϕ-R2096 on carbapenem-resistant *A. baumannii* YMC13/03/R2096 strain. The host bacterium, *A. baumannii* YMC13/03/R2096, was infected with phage Bϕ-R2096 at MOIs of 0.1, 1, and 10. The turbidity of the bacterial cultures was measured by spectrophotometer at OD_600_. Data are presented as the mean ± standard deviation

The bacteriolytic effect of phage Bϕ-R2096 on the *A. baumannii* YMC13/03/R2096 isolate in vitro is shown in Fig. 2. The absorbance (OD_600_) of the uninfected control culture increased rapidly (OD_600_ = 1.49, 6 h), whereas all phage-Bϕ-R2096-infected cultures showed significantly inhibited bacterial growth after 3 h (MOI = 10, OD_600_ = 0.37, 6 h), although the bactericidal effect exhibited slight differences with changes in the MOI (Fig. 2).

### Genome sequencing and bioinformatics analysis

**Fig. 3:**
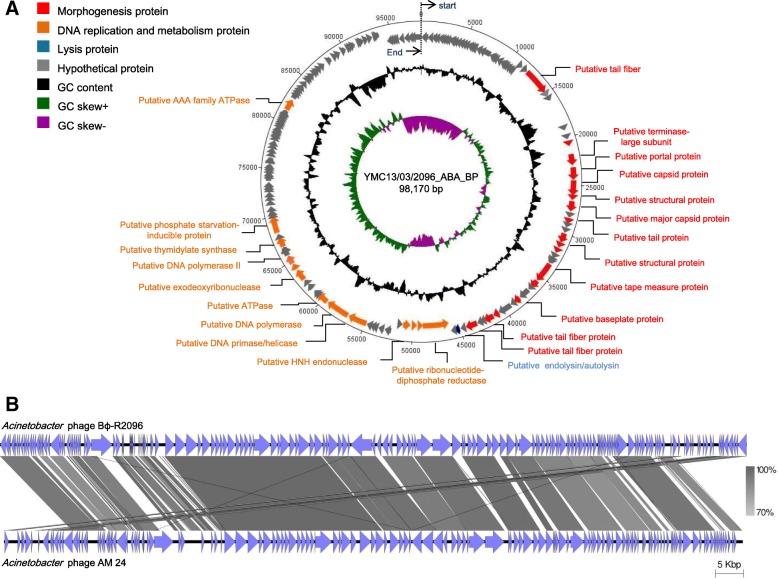
Circular map of the *A. baumannii* phage Bϕ-R2096 genome (**a**) and genome comparison between *A. baumannii* phage Bϕ-R2096 and phage AM24 (**b**). Circular map of the *A. baumannii* phage Bϕ-R2096 prepared using DNAPlotter (**a**). The outer arrows present ORFs of phage Bϕ-R2096 and the direction of transcription. The GC content appears in the black ring, and the inner rings are GC skew+ (+, green; −, purple). Genomes of *A. baumannii* phage Bϕ-R2096 and phage AM24 were compared using Easyfig software (**b**). Homology (blastn) is indicated between gray lines for each phage genomes and putative ORFs with direction of transcription are presented as arrows

The genome of phage Bϕ-R2096 was sequenced with 58,755 read lengths and 285-fold coverage. The linear dsDNA of phage Bϕ-R2096 was illustrated in Fig. 3a as a circular form using DNAPlotter. The phage Bϕ-R2096 genome was annotated 32 of the 162 ORFs in the phage Bϕ-R2096 genome, and among them, 12 predicted proteins for phage structures (*orf* 41, *orf* 43, *orf* 44, *orf* 46, *orf* 51, *orf* 55, *orf* 56, *orf* 62). The DNA metabolism modules (*orf* 81, *orf* 87, *orf* 88, *orf* 89) exhibited 24–51% protein sequence similarity with *A. baumannii* phage vB_AbaM_Acibel004 (GenBank accession no. NC_025462) in the BlastP database (Additional file 1: Table S2). Also, the putative tail fiber (*orf* 34) and putative endolysin (*orf* 68) revealed 71 and 56% of sequence similarity with *A. baumannii* phage AM24 and *A. baumannii* phage AP22, respectively (Additional file 1: Table S2). However, the whole genome of the novel phage Bϕ-R2096 has no BlastN matches with any other *Acinetobacter* phage genomes, except for *Acinetobacter* phage AM24 (GenBank accession number KY000079). As shown in Fig. 3b, the genomic structure of phage Bϕ-R2096 was compared with that of phage AM24. This alignment indicates that the gene organization of phage Bϕ-R2096 and phage AM24 are similar, and share an overall high DNA sequence similarity (the query coverage of 81%, the max identity of 98%). The virulence and lysogeny-related genes (encoding protein such as integrase) were not detected in the phage Bϕ-R2096 genome. In phage Bϕ-R2096 genome, 239 promoters and 45 rho-independent transcription terminators were predicted by using BPROM and ARNold software, respectively.

### Therapeutic effect of phage Bϕ-R2096 against CRAB in *Galleria mellonella* infection model

**Fig. 4:**
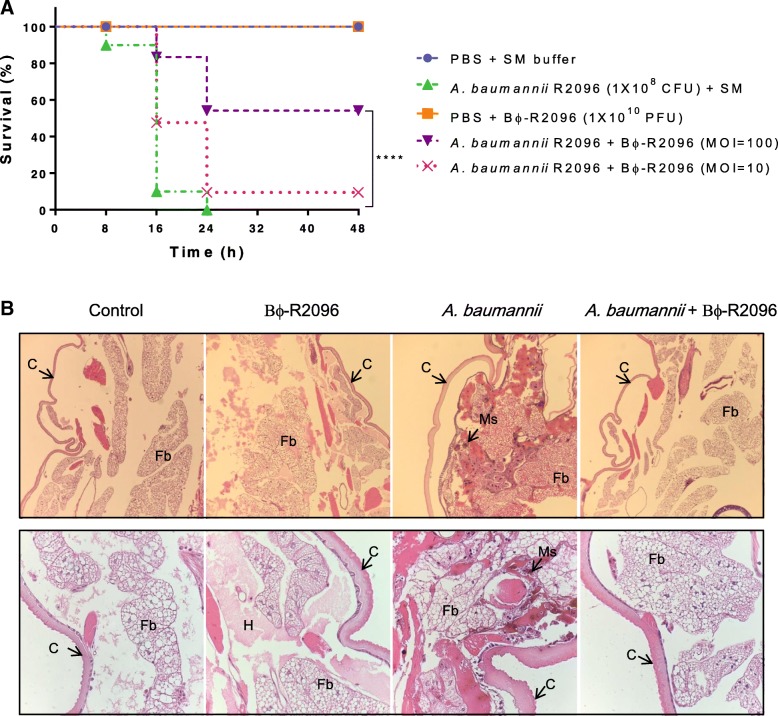
Survival of *G. mellonella* larvae treated with *A. baumannii* phage Bϕ-R2096 against carbapenem-resistant *A. baumannii* YMC13/03/R2096 (**a**) and histological features of the *G. mellonella* larvae (**b**). *G. mellonella* larvae treated with *A. baumannii* phage Bϕ-R2096 (MOI 100 and 10) after infection with CRAB YMC13/03/R2096, including the survival of each larval group, were monitored at 8-h intervals for 48 h. Tissue sections of larvae from each group were stained at 24 h with hematoxylin and eosin and observed at a magnification of × 10 (upper column) and × 40 (down column). C: cuticle; Fb: fat body; H: hemolymph; Ms.: melanized structures. The percentage of *G. mellonella* survival at each time point is presented using the log-rank (Mantel-Cox) test (GraphPad Prism v 5.03). The results show the average of three independent trials (*n* = 20 larvae per group). Log-rank (Mantel-Cox) test, ****p < 0.0001

We used *Galleria mellonella* larvae as an animal model to evaluate the effectiveness of *A. baumannii* phage Bϕ-R2096 as a therapy against the YMC13/03/R2096 strain of CRAB. *G. mellonella* larvae were treated with concentrated phage Bϕ-R2096 (1 × 10^10^ PFU) at two MOIs (MOI 100 and 10) 30 min after infection with CRAB (1 × 10^8^ CFU). The results in Fig. 4a show that the bacteria-only-infection group died rapidly: 90 and 100% of larvae were dead at 16 h and 24 h, respectively. However, the postinfection phage-treatment larval group at an MOI of 100 had a survival rate of 80 and 50% at 16 h and 48 h, respectively. There was a statistically significant improvement in survival rates of larvae treated with phage at MOI of 100 and untreated control larvae (*p* < 0.0001) at 48 h. The phage-treated larval group at an MOI of 10 exhibited 45% survival at 16 h, but this group showed only 10% survival at 48 h. The two groups injected with buffer (PBS + SM) and concentrated phage solution (1 × 10^10^ PFU) showed no mortality or signs of melanization after 48 h; thus, the phage caused no virulence, and the injection caused no trauma.

To observe the effects of phage therapy in the larval tissue, we examined the histology of larvae from each experimental group. As shown in Fig. 4b, many melanized nodules were detected in various areas of larval tissue from the bacteria-only-treatment group (1 × 10^8^ CFU); however, the larval group that received postinfection phage treatment (MOI 100) had significantly less tissue damage and melanization in the fat body well and the muscle layer than the group that received only bacteria. Furthermore, the phage-only-treatment (1 × 10^10^ PFU) groups did not exhibit any tissue damage not also seen in the buffer-only-treatment (PBS + SM) group.

### Therapeutic effect of phage against CRAB in a mouse model of acute pneumonia

**Fig. 5:**
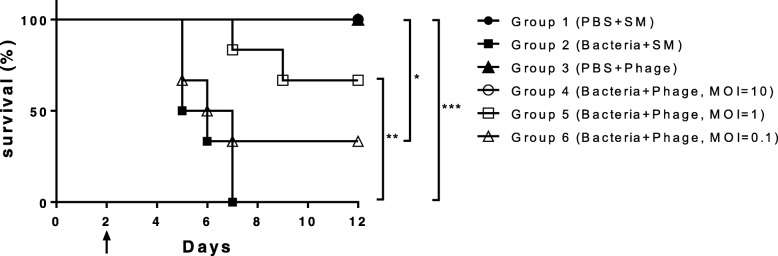
Therapeutic effects of *A. baumannii* phage Bϕ-R2096 in a mouse model of acute pneumonia caused by carbapenem-resistant *A. baumannii* YMC13/03/R2096 strain. The C57BL/6 mice were intranasally inoculated with phage, bacteria, or both (*n* = 6 mice per group). Group 1 (control): PBS + SM buffer treatment; Group 2: *A. baumannii*-only-treatment; Group 3: phage Bϕ-R2096-only-treatment; Groups 4–6: postinfection phage Bϕ-R2096-treatment (MOI 10, 1, 0.1) 30 min after *A. baumannii* infection. A second cyclophosphamide (CP) and first bacteria treatment was given on day 2 (black arrow). Log-rank (Mantel-Cox) test, ***p < 0.001, *p = 0.0236

We investigated the survival rate in a mouse model of acute pneumonia to assess the therapeutic effect and safety of *A. baumannii* phage Bϕ-R2096 as an antibacterial agent. As shown in Fig. 5, *A. baumannii* phage Bϕ-R2096 exhibited excellent elimination of the target bacteria. The bacteria-only-treatment mouse group all died by day 5 postinfection; however, the mouse groups who received phage treatment at 30 min postinfection showed high survival rates on day 12 at MOI = 10 (100%), MOI = 1 (60%), and MOI = 0.1 (30%). Moreover, no mice in the phage-only group or the control group (buffer-treated) died and lost weight (Additional file 1: Figure S4).

### Histological changes and cytokines

**Fig. 6:**
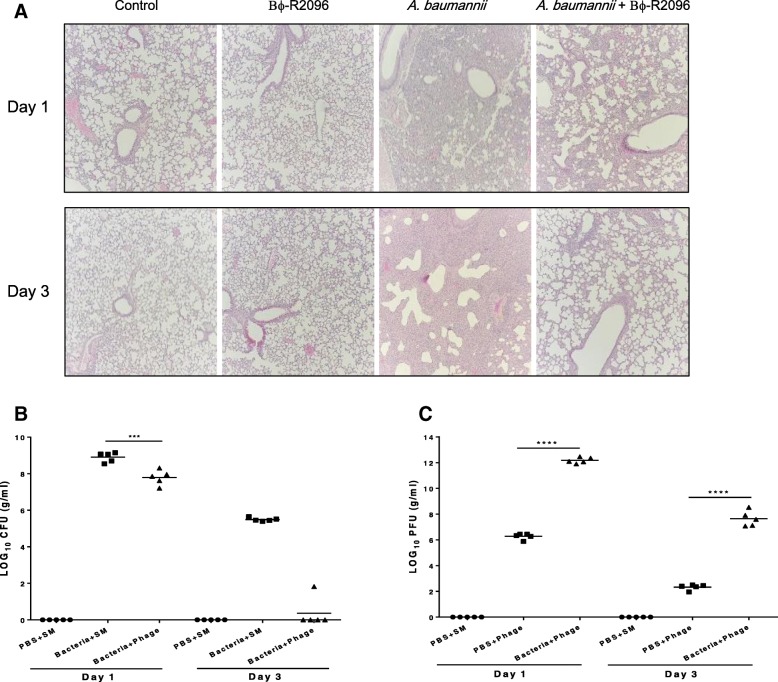
Histological features of mouse lungs (**a**), bacteria CFUs (**b**), and phage PFUs (**c**) in mouse lungs on days 1 and 3 after treatment with carbapenem-resistant *A. baumannii* YMC13/03/R2096 clinical strain, phage Bϕ-R2096, or both. Five mice were sacrificed from each group on days 1 and 3. Sections of mouse lungs were stained with hematoxylin and eosin and observed at a magnification of × 10. The horizontal bar represents the mean value for each group. The one-way ANOVA with Tukey’s multiple comparisons test was used to compare the phage concentration data. Significant differences (****p < 0.0001) are indicated by asterisks

We used a histological analysis and the immunogenicity of lung samples to evaluate the efficacy of the phage therapy in the mouse acute pneumonia model. We compared the histological changes in the lung tissues from each group using H&E staining (Fig. 6a). The bacteria-only-treatment group sustained significant damage, such as severe thickening of the alveolar walls and hemorrhaging in the alveolar space, on days 1 and 3, but the group that received phage treatment postinfection showed only a slight region of mild or moderate alveolar wall thickening. Moreover, the group that received only phages showed no histological changes compared with the control (buffer-treated) group on days 1 and 3. In the cytokine analysis (TNF-α, IL-10, IL-1β), the levels of TNF-α (****p < 0.0001), IL-6 (****p < 0.0001), except IL-1β (no significant difference), in the lungs of the postinfection phage-treatment group were significantly reduced compared with the bacteria-only-treatment group on day 1. The phage-only-treated group and control (buffer-treated) group exhibited no appreciable levels of TNF-α, IL-6 or IL-1β (Additional file 1: Figure S5).

### Bacterial clearance and phage count

We measured the number of bacteria and phages in mouse lungs from each group on days 1 and 3. Each point of Fig. 6 (b) and (c) indicates the bacterial or phage counts of a single mouse. The bacterial load in the lungs of the postinfection phage-treatment group declined > 2 log_10_ CFU (***p < 0.001) compared with the bacteria-only-treatment group on day 1, and in the postinfection phage-treatment group, bacteria were cleared completely from most of the mouse lungs on day 3 (Fig. 6b). Viable bacteria were not detected in the buffer only and phage-only-treatment groups (data not shown) at the same time points. In phage counts in the mouse lungs, the PFU value in the postinfection phage-treatment group was significantly higher (> 6 log_10_ PFU, ****p < 0.0001) than in the phage-only-treatment group on day 1. By day 3, the number of phages in the postinfection phage-treatment group had decreased significantly (12.2 to 7.7 log_10_ PFU), and the PFU value of the phage-only-treatment group had declined from 6.2 to 2.3 log_10_ PFU (Fig. 6c). In this test, the control (buffer-treated) group and the bacteria-only-treatment group (data not shown) had no detectable phage plaques at any time point.

## Discussion

The development of new antibiotics and their dramatic effect on bacterial diseases have improved human life since the middle of the twentieth century, but unfortunately the emergence and worldwide spread of MDR bacteria has become a major global challenge [52].

MDR bacteria are resistant to at least three different classes of antibiotics, such as carbapenems, aminoglycosides, and quinolones [53]. MDR *Acinetobacter* spp*.*, including CRAB, have been increasing during the past decade and are of great medical concern worldwide [22]. Because the need to control MDR infections is urgent, phages are being newly studied as potential antibiotic alternatives. Phage-related therapy has unique advantages, including high specificity to target pathogens, self-replication, and low toxicity, compared with commercial antibiotics [30, 35], which explains why the therapeutic effects of various phages against many MDR bacterial infections, such as *Staphylococcus aureus*, *P. aeruginosa*, and *E. coli*, have been widely studied in animals [54–57].

In 2006, the US FDA approved food applications of phages to eliminate food-borne pathogens such as *Listeria monocytogenes*, *Salmonella* spp., and *Shigella* spp. [35, 58]. Thus, bacteriophages are safe in humans and stable in the environment, so they can be applied as alternative therapeutic agents.

The purpose of this study was to characterize novel *Acinetobacter* phage Bϕ–R2096, which lyses MDR *Acinetobacter* spp*.* including CRAB clinical isolates, in detail and to provide in vivo data on the therapeutic effects of phage Bϕ-R2096 against CRAB clinical isolates in *G. mellonella* infection and mouse acute pneumonia models.

In this study, the host bacterium, CRAB YMC13/03/R2096 strain, isolated from a patient is an ST 357 strain belonging to EU clone II [59]. Especially, 16S rRNA methylase ArmA gene which has high level of resistance to various aminoglycosides also was identified in this organism (data not shown) [60].

The morphological features of phage Βϕ-R2096 indicate that it is part of the family *Myoviridae*; it is similar to other *Myoviral Acinetobacter* phage AP22 [61] and vB_AbaM-IME-AB2 [40] (Fig. 1). The phage Βϕ-R2096 formed clear round plaques, and showed plaque-surrounding halos which are associated with phage-derived depolymerases on agar plates. This phenomenon indicates the presence of depolymerase activity which degrades capsular exopolysaccharides producing bacteria [62].

In vitro characterization of phage Βϕ-R2096 showed that it has a high adsorption rate and burst size (Additional file 1: Figure S2). In particular, this phage was relatively stable at high temperatures and more stable at an alkaline pH than an acidic pH for up to 10 months (Additional file 1: Figure S3). These results suggest that phage Βϕ-R2096 is more stable for long periods in storage and various physiological conditions than that of previous reports [39, 63, 64]. In the host spectrum using spot test, phage Βϕ-R2096 exhibited a specific and broad host range on the ST357 CRAB strains. Especially; efficiency of plating (EOP) assay was performed to assess a quantitative measure and possible “lysis from without” phenomenon of lytic activities of phage Βϕ-R2096 against CRAB strains (Table 1) [65]. In the host cell lysis test, phage Βϕ-R2096 strongly prevented bacterial growth dose dependently in vitro (Fig. 2). This result indicates that phage Βϕ-R2096 is a CRAB-specific lytic phage that could be a promising antimicrobial agent to control CRAB.

To date, approximately 27 *A. baumannii* phage genomes have been completely sequenced and deposited in the NCBI database (http://www.ncbi.nlm.nih.gov/genome/, May 1, 2018), and recently, the potential of therapeutic phage as a biocontrol agent against MDR-*A. baumannii* has been reported [44, 66–71]. Zhou W et al. [66] and Mathias Jansen et al. [67] presented synergy of antibiotics and phages for the control of *A. baumannii* strains in in vitro or in vivo, and Regeimbal JM et al. [44] and Yin S et al. [68] also stated the therapeutic efficacy of phage against *A. baumannii* using mouse wound infection model. LaVergne S et al. [69] attempted human trial on a patient with the MDR-*A. baumannii* craniectomy site infection. However, there is little information studying the efficacy of phage therapy in the *G. mellonella* bacteremia and the mouse lung infection with CRAB clinical isolates. Jeon J et al. [71] and Yunfen Hua et al. [70] reported that in the intranasal treatment of monophage, phage therapy rescues the mice from lung infection caused by CRAB strains. In this study, we also investigated in details a novel *Acinetobacter* phage Bϕ-R2096 against CRAB clinical isolates in vivo and in vivo. In particular, to evaluate the therapeutic efficacy of phage, we employed the *G. mellonella* wax moth larvae infection and the mouse acute pneumonia model.

In a *G. mellonella* infection model, a single dose of phage Bϕ-R2096 increased survival rate of *G. mellonella* against CRAB clinical isolates. Moreover, the *G. mellonella* used in this study exhibited no toxicity from the concentrated phage (1 × 10^10^ PFU) injections.

In a previous study, at low MOI, survival of *G. mellonella* was similar to that of our phage for 20 h, but these *G. mellonella* larvae were treated with phage cocktail [72]. In another study, although *G. mellonella* were treated with MOI of 0.1 showed higher survival of larvae than this study [66]; however, we used CRAB strain which includes the 16S rRNA methylase gene armA clinical strain as host in this study.

Previous in vivo studies of the therapeutic potential of phages against bacterial pathogens such as *P. aeruginosa*, *Clostridium difficile*, *Klebsiella pneumonia*, and *A. baumannii* have used a *G. mellonella* infection model [44, 48, 50, 73, 74]. Also, some studies have shown a significant correlation between the *G. mellonella* model and a mouse infection model, moreover, phages improved survival in a dose dependent and time-dependent manner in these infection models; in fact, bacterial isolates are more virulent in the *G. mellonella* larval model than they are in the mouse model [74, 75]. Thus, compared with the mouse infection model, the *G. mellonella* larval model is a simpler, faster, more cost-effective, and more predictive model system for studying both the toxicity of pathogens and the therapeutic effects of phages against bacterial infections [74].

In the mouse acute pneumonia model, a single dose of phage Bϕ-R2096 produced a strong therapeutic effect in all the mice. Especially, this phage exhibited approximately 2 to 3-fold higher survival rate at low MOI (MOI of 0.1) than that of Jeon J et al. [71] and Yunfen Hua et al. [70]. To investigate whether high-dose intranasal phage administration had side effects in this mouse model, we intranasally administered a high dose of phage Bϕ-R2096 (10^11^ PFU/ml) to female C57BL/6 mice (*n* = 6). No deaths, decreases in bodyweight, or abnormal symptoms, such as lethargy, piloerection, or hunching occurred during the following 15 days (data not shown). Also, none of those animals (n = 6) exhibited apparent histological changes in their lungs (data not shown). Therefore, the intranasal administration of a single high dose of phage Bϕ-R2096 had no significant side effects on the health of the animals in the mouse infection model. Our results thus show that phage Bϕ-R2096 can eliminate MDR pathogens and ameliorate disease symptoms in animals without causing any adverse effects.

In view of the correlation between the two in vivo models: although the two models used different infection routes for the phage and bacteria, and the larval model did not show survival rates as high as those in the mouse infection model, both models showed improvements from the phage treatment. Therefore, we suggest that in future studies, the *G. mellonella* model is an adequate animal model for assessing the safety and effectiveness of phage therapy.

## Conclusions

*Acinetobacter* phage Βϕ-R2096 is a newly discovered *Myoviral* bacteriophage, and we investigated its physiological characteristics and performed a whole genome analysis. Also, we evaluated its therapeutic effects against the carbapenem-resistant *A. baumannii*YMC13/03/R2096 clinical strain in two animal infection models.

Overall, phage Bϕ-R2096 showed a strong bacteriolytic activity in vitro and a significant reduction in mortality in both the *G. mellonella* larval model and the mouse acute pneumonia model; moreover, it ameliorated the pathogenic effects of the CRAB infection in both wax-moth larvae and mouse lungs. Interestingly, the two animal models also showed significant correlation for the efficacy of the phage as a therapeutic agent. In this study, our research strongly suggests that phage treatment can effectively eliminate pathogens and reduce the mortality of CRAB infections in vitro and in vivo. Therefore, we expect that phages will become new therapeutic agents for treating human pulmonary infections caused by clinical CRAB. Furthermore, the clear understanding of the physiological and molecular features of phages that we provided in this study proposes new promising strategies to control MDR pathogens. To the best of our knowledge, this is the first study to compare the therapeutic efficacy of a phage lysing CRAB between the *G. mellonella* infection model and the mouse acute pneumonia model.

## Methods

### Bacterial strains

The 20 CRAB strains used in this study to screen *A. baumannii* lytic phages were collected from patient samples taken at a tertiary-care hospital in Korea in 2013. The identification and antimicrobial susceptibility by CLSI guidelines of the CRAB clinical isolates were confirmed using previously published methods [76]. We selected 20 carbapenem-resistant and 11 carbapenem-sensitive *A. baumannii* clinical isolates for the host spectrum test of the isolated phage. We used pulsed-field gel electrophoresis (PFGE) to analyze bacterial genetic differences (Additional file 1: Figure S1). To describe the genetic backgrounds of the CRAB isolates, we conducted multilocus sequence typing (MLST), and we used a multiplex PCR assay to detect OXA carbapenemase genes in the CRAB strains [77]. The list of bacterial strains used in this study is given in Table 1. The carbapenem-resistant *A. baumannii* YMC13/03/R2096 strain from the sputum of a patient with pneumonia, the host bacteria of phage Bϕ-R2096, was used to study the preparation, physiological characteristics, and in vivo therapeutic effects of phage Bϕ-R2096. Eleven carbapenem-susceptible *A. baumannii*, three colistin-resistant *A. baumannii*, three carbapenem-resistant *Pseudomonas aeruginosa*, and three carbapenem-resistant *Escherichia coli* were used to determine the infectivity of phage Bϕ-R2096.

### Isolation of bacteriophage

The CRAB lytic phages isolated from sewage samples of a hospital in South Korea were purified and concentrated using methods described previously [76, 78]. Briefly, the sewage water was treated NaCl (1 M) and polyethylene glycol (PEG) 8000 (total volume of 10%, Sigma), incubated at 4 °C for 24 h, filtered using 0.22 μm membranes (Millipore Corporation, Bedford, MA, USA), and then centrifuged at 12,000×g for 1 h at 4 °C and resuspended in sterilized sodium chloride-magnesium sulfate (SM) buffer (100 mM NaCl, 8 mM MgSO_4_, 2% gelatin, 50 mM Tris–HCl, pH 7.5). The resuspended solution was mixed with 20 *A. baumannii* strains and incubated at 37 °C for 24 h, and then the cultured solution was centrifuged and filtered. For initial phage isolation, spot tests were performed, and one single-clear plaque of the formed plaques by double-layer agar method was transferred in to a tube of LB broth using a sterile pipette tip. For phage purification, this process was repeated until one-plaque morphology was exhibited at least three times [61]. To concentrate the purified phages, we precipitated them using PEG 8000 (total volume of 10%), centrifuged them (12,000×g at 4 °C for 1 h), and then resuspended them in SM buffer. Phage titration was calculated by the plaque assay using a double-layer method [78].

### Transmission electron microscopy

Concentrated phage Βϕ-R2096 (approximately 10^11^ PFU/ml) was adsorbed onto carbon-coated copper grids and negatively stained with 2% uranyl acetate for 15 s. Phage morphologies were confirmed using a transmission electron microscope (JEOL JEM-101, Tokyo, Japan) at 80 kV.

### Host range test

The host range of the purified phage against the collected clinical isolates was determined by spot tests, as described previously with some modifications [79]. Briefly, purified phage stock (1 × 10^10^ PFU/ml) was serially diluted with SM buffer. 5 μl drop of diluted phage solution was spotted and dried on Luria-Bertani (LB) agar plates, each of which contained a different bacterial strain, and then the plates were incubated at 37 °C for 12 h. When clearing zones formed against each bacterial host, plaque clarity was evaluated as clear (++), turbid (+), and no plaque (−). Efficiency of plating (EOP) was evaluated using diluted phage suspension (1 × 10^5^ PFU/ml) by the double-layer agar plate method, and was presented by the ratio of phage titer on the test strain to that on the host strain [80].

### Host cell lytic activity test

The host bacterium, *A. baumannii* YMC13/03/R2096, was cultured up to OD_600_ = 0.2 at 37 °C in 30 ml LB medium and mixed with the phage at a multiplicity of infection (MOI) of 0.1, 1, and 10. During shake-culturing at 37 °C, samples of 1 ml were taken at 1 h intervals for 6 h, and bacterial turbidity was measured by spectrophotometry at OD_600_ nm; these tests were assessed in triplicate.

### Genome sequencing and bioinformatics analysis

Bacteriophage genomic DNA was extracted using standard phenol–chloroform extraction protocols, as described previously [81]. The genome sequencing of purified phage DNA was conducted at ChunLab, Inc. (Seoul, South Korea) using a 454 GS Junior Genome analyzer (Roche Life Sciences, Branford, CT, USA). The complete genome sequence was analyzed using the Roche gs Assembler (version 2.6; Roche) and CLC genomics wb 4.8 (CLCbio, Aarhus, Denmark). We compared it with the genome sequences of other phages using the NCBI database (http://www.ncbi.nlm.nih.gov/). The prediction of open reading frames (ORFs) was performed using the NCBI ORF finder and GenMark.hmm software [82]. The putative promoter and rho-independent transcription terminators were predicted by using the Softberry program (http://www.softberry.com) and ARNOLD software (http://rna.igmors.u-psud.fr/toolbox/arnold/), respectively. The tRNA genes were predicted by using the tRNAscan-SE program [83]. The similarities (blast E value cutoff of 0.1) of all putative proteins were confirmed by BlastP and PSI-BLAST (http://www.ebi.ac.uk/Tools/sss/fasta/). A map of the annotated phage genome was generated using DNAPlotter [84], and phage genome was compared by using Easyfig software (version 2.1) [85].

### *Galleria mellonella* larvae infection model

*Galleria mellonella* wax moth worms were used as an in vivo model to assess the therapeutic effects of the isolated *A. baumannii* lytic phage against *A. baumannii* clinical strains and were evaluated as described previously, with some modifications [86].

All the *G. mellonella* larvae were maintained on an artificial diet (25% liquid honey, 20% glycerin, 5% dried beer yeast, 15% wheat flour, 15% skim milk powder and 20% polent) for 2 days at 25 °C. And larvae kept without food in a 90-mm Petri dish in darkness for 24 h at 37 °C before the experiments. Wax moth worms were randomly selected (weight 200–250 mg) and swabbed with 70% ethanol to reduce potential contamination caused by the injection. Larvae were divided into 5 groups: 1. Buffer (phosphate-buffered saline [PBS] + SM)-only group, 2. Bacteria-only-treatment (1 × 10^8^ CFU/ml) group, 3. Phage-only-treatment group (1 × 10^10^ PFU/ml), 4. Postinfection phage (MOI 100)-treatment group (1 × 10^10^ PFU/ml), and 5. Postinfection phage (MOI 10)-treatment group (1 × 10^9^ PFU/ml). Thirty minutes after larvae received 5 μl of bacteria in the right side last proleg by injection, 5 μl of phages or buffer were injected into a different last proleg using a 10-μl Hamilton syringe (701RN; Hamilton Bonaduz AG, Bonaduz, Switzerland). The injected larvae were incubated in the dark at 37 °C in 90-mm plastic Petri dishes and monitored for their survival at 8-h intervals for 48 h. *Galleria mellonella* larvae were considered dead when they did not move in response to touch with a pipette tip. All experiments used 10 larvae per group and were repeated three times.

### Histology of larvae

The collected larvae were processed for histology, as previously described with modifications [87]. Briefly, the larvae in each group were fixed in 10% formalin for 4 days (injected with 100 μl of 10% formalin) and embedded in paraffin. The larval tissue sections were routinely stained with hematoxylin and eosin (H&E), and the tissue morphology was observed using an optical microscope.

### Phage therapy in the mouse infection model

To evaluate the therapeutic safety and efficacy of the bacteriophage in vivo, we used six groups of C57BL/6 mice (female aged 7–8 weeks) with six mice per group, divided as follows: group 1, buffer-only-treatment (PBS, Invitrogen, + SM) mouse group; group 2, bacteria-only-treatment mouse group; group 3, phage-only-treatment mouse group; group 4–6, postinfection phage-treatment (MOI = 10, 1, 0.1) mouse groups. Briefly, all of the mice used in the experiments were immunized by the intraperitoneal (i.p.) route using cyclophosphamide (200 mg/kg, Sigma-Aldrich) at 48-h intervals [88]. Mice were treated by the intranasal route with phage solution (1 × 10^10^ PFU/ml, 30 μl) or SM buffer 30 min after infection with 1 × 10^9^ CFU/ml, 30 μl, or PBS buffer administration by i.p. injection while anesthetized with Zoletil-Rompun. Each group was monitored for mortality, abnormal behavior, and body weight for 12 days.

For bacterial clearance, phage count, cytokine, and histology analyses, four groups of mice (ten mice per group) were divided as follows: group 1, buffer-only-treatment (PBS and SM) mouse group; group 2, phage-only-treatment mouse group; group 3, bacteria-only-infection mouse group, and group 4, postinfection phage-treatment (MOI = 10) mouse group (30 min after infection). These mice did not receive cyclophosphamide injections. Mice were sacrificed on day 1 (five mice per group) or 3 (five mice per group) after measuring their body weights, and their lungs were collected. Blood was sampled from the eyes of the mice at the same time. The supernatants of the lung lysates and serum were stored at − 70 °C for the cytokine analysis. The mice were euthanized through CO_2_ asphyxiation followed by cervical dislocation.

### Bacteria clearance and phage counting

To determine bacterial counts in the mouse lungs at days 1 and 3 after bacterial infection, the lung samples from each group were homogenized and serially diluted in PBS and then plated onto LB agar plates with ampicillin (50 μg/ml). To assess the bacteriophages, the supernatants of the lung and blood samples collected from each mouse group were serially diluted and counted using the double-layer agar method at each time point.

### Histology of mouse lungs

Mouse lungs were processed for histology, as previously described with modifications [89]. Briefly, to evaluate histological features, the right lung tissues were removed and fixed in 10% formalin. The specimen were dehydrated in graded alcohol and embedded in paraffin. The lung tissue sections of 3 μm-thick were stained with H&E and observed using an optical microscope.

### Ethics statement

All animal experiments followed the regulations of the Institutional Animal Care and Use Committee of Yonsei University College of Medicine, Seoul, Korea (IACUC Approval no. 2014-0031-2).

### Statistical analysis

We used the log-rank (Mantel-Cox) test and statistical software (GraphPad Prism Software, version 6; GraphPad Software, San Diego, CA, USA) to compare groups in the survival curve test. A one-way ANOVA followed by Tukey’s test (GraphPad Prism Software) was used to compare statistical calculations for bacterial and phage titers.

## Additional file

Additional file 1: **Figure S1.** Pulsed-field gel electrophoresis (PFGE) analysis of 31 carbapenem-resistant and -susceptible *A. baumannii* isolates. **Figure S2.** The adsorption rate (A) and one-step growth curve (B) of *A. baumannii* phage Bϕ-R2096 on *A. baumannii* strain YMC13/03/R2096. **Figure S3.** Temperature and pH stability of *A. baumannii* phage Bϕ-R2096 on *A. baumannii* strain YMC13/03/R2096. **Figure S4.** Body weight of C57BL/6 mice infected with *A. baumannii* phage Bϕ-R2096, *A. baumannii* YMC13/03/R2096, or both. **Figure S5.** The concentration of cytokines (TNF-α, IL-6, and IL-1β) in the lungs of mice on days 1 and 3 after treatment with *A. baumannii* YMC13/03/R2096, phage Bϕ-R2096, or both. **Table S1.** Antibiotic resistance profiles of carbapenem-resistant *A. baumannii* clinical strains used in this study. **Table S2.** *A. baumannii* phage Bϕ-R2096 ORFs summary. (DOCX 698 kb)

## Abbreviations

CFU: Colony forming units
CRAB: carbapenem-resistant *Acinetobacter baumannii*
EOP: Efficiency of plating
ESKAPE: *Enterococcus faecium*, *Staphylococcus aureus*, *Klebsiella pneumoniae*, *Acinetobacter baumannii*, *Pseudomonas aeruginosa*, and *Enterobacter* species
MLST: multilocus sequence typing
MOI: Multiplicity of infection
PFGE: pulsed-field gel electrophoresis
PFU: Plaque forming units
