# Supplementary material for: Efficacy of bacteriophage treatment against carbapenem-resistant Acinetobacter baumannii in Galleria mellonella larvae and a mouse model of acute pneumonia
Source: BMC Microbiol. 2019 Apr 2;19:70. doi: 10.1186/s12866-019-1443-5 (PMC6444642; doi:10.1186/s12866-019-1443-5)
Supplement: Supplementary file 1 — Figure S1. Pulsed-field gel electrophoresis (PFGE) analysis of 31 carbapenem-resistant and -susceptible A. baumannii isolates. Figure S2. The adsorption rate (A) and one-step growth curve (B) of A. baumannii phage Bϕ-R2096 on A. baumannii strain YMC13/03/R2096. Figure S3. Temperature and pH stability of A. baumannii phage Bϕ-R2096 on A. baumannii strain YMC13/03/R2096. Figure S4. Body weight of C57BL/6 mice infected with A. baumannii phage Bϕ-R2096, A. baumannii YMC13/03/R2096, or both. Figure S5. The concentration of cytokines (TNF-α, IL-6, and IL-1β) in the lungs of mice on days 1 and 3 after treatment with A. baumannii YMC13/03/R2096, phage Bϕ-R2096, or both. Table S1. Antibiotic resistance profiles of carbapenem-resistant A. baumannii clinical strains used in this study. Table S2. A. baumannii phage Bϕ-R2096 ORFs summary. (DOCX 698 kb) [file 12866_2019_1443_MOESM1_ESM.docx]

**Supplementary Material**

**Efficacy of bacteriophage treatment against carbapenem-resistant *Acinetobacter baumannii* in *Galleria mellonella* larvae and a mouse model of acute pneumonia**

**1. Supplementary Methods**

**Phage adsorption and one-step growth kinetic test**

The adsorption rate and one-step growth kinetics of phage Bϕ-R2096 were performed as previously described with some modification  [1, 2]. For the adsorption rate test, the host bacterial suspension (10^5^ CFU/ml) was mixed with the phage solution at MOI=0.01 and incubated at 37°C. Samples of the mixture (100 μl) were taken at 1, 2, 3, 4, 5, and 10 min and centrifuged immediately (12,000×g, 10 min). The supernatants were used determine the titer of unadsorbed phages using the double-layer agar plate method. For the one-step growth curve test, 40 μl of phage solution (10^4^ PFU/ml) was added to 4 ml of bacterial suspension (10^5^ CFU/ml) and incubated at 37°C for 5 min. The mixture was centrifuged (12,000×g, 10 min), and the supernatant, which included the unadsorbed phages, was removed. Then the pellets were resuspended in fresh LB medium of the same volume. Subsequently, culture samples were taken every 5 min for 120 min. The samples were immediately diluted and plated using the double-layer agar plate method. The agar plates were incubated for phage titration overnight at 37°C.

**Thermal and pH stability testing**

The thermal and pH stability of the phage were determined using the double-layer agar method  [3]. To evaluate the thermal stability, a concentrated phage solution (1.7×10^9^ PFU/ml) was incubated at 25°C, 40°C, 50°C, 60°C, and 70°C for 3 h, 6 h, and 9 h. To evaluate the pH stability, buffers at pH 4, 5, 6, 7, 7.5, 8, 9, and 10 were used, and the phage titer was measured on days 1, 3, 6, 1 month, and 10 months. The initial control titer (4°C, pH 7.5) was used for comparison with each sample titer, and the data are represented as a percentage of the phage titer (PFU/ml).

**Measurement of cytokines**

The level of tumor necrosis factor alpha (TNF-α) interleukin 6 (IL-6), and interleukin 1β (IL-1β) in lung lysates was measured using the commercial DuoSet kit (R&D Systems, Inc., USA) as specified by the manufacturer.

**2. Supplementary Figures and Table**


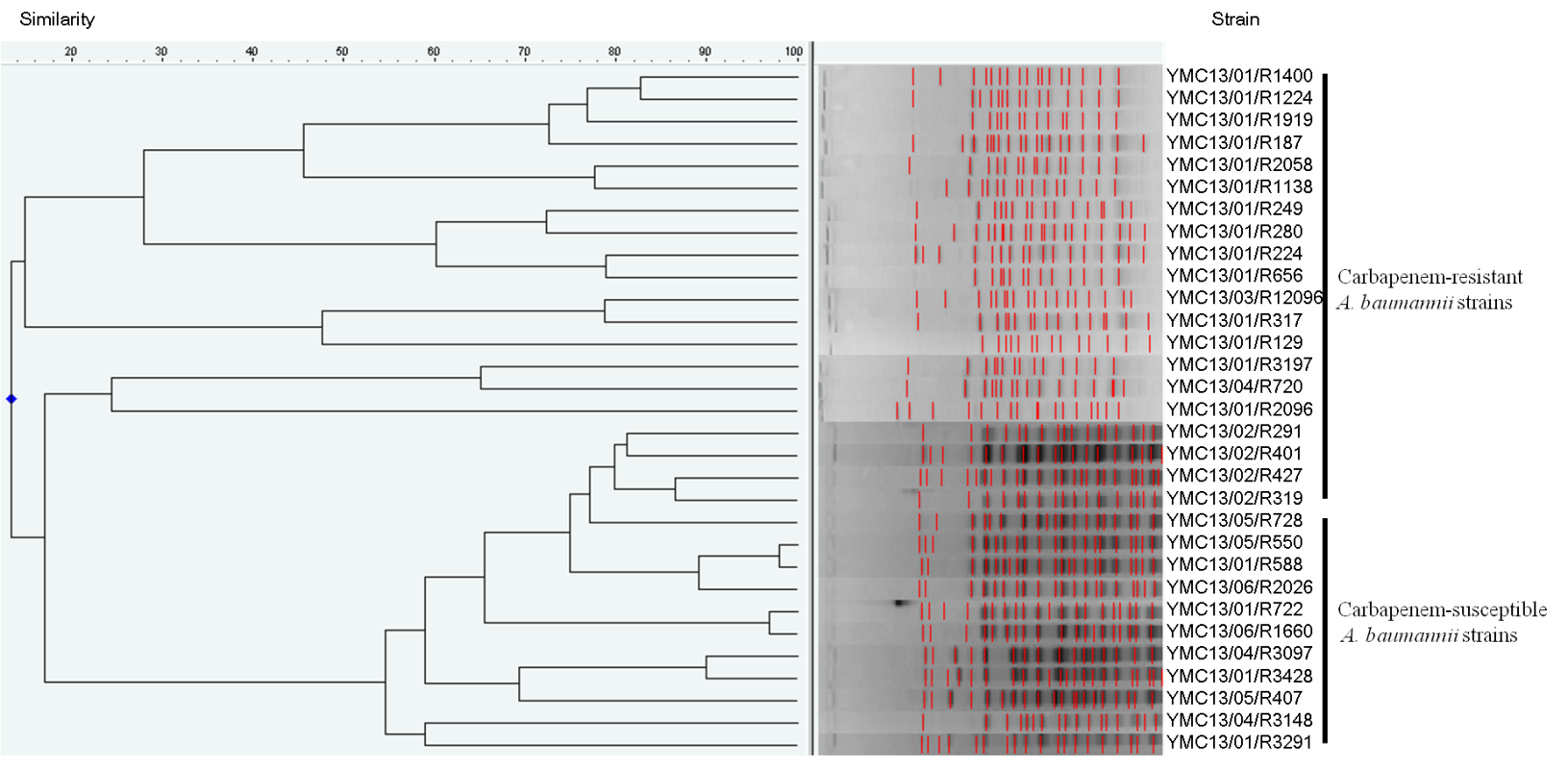


Figure S1. Pulsed-field gel electrophoresis (PFGE) analysis of 31 carbapenem-resistant and -susceptible *A. baumannii* isolates. *Xba*I-restricted DNA from *A. baumannii* isolates was separated by PFGE using a CHEF-DR II system (electrophoresis conditions: 1% agarose; 6 V/cm; high 10 s and low 3 s in a linear manner for 20 h; Bio-Rad, Hercules, CA). The similarity dendrogram of the PFGE patterns of *A. baumannii* isolates was analyzed using InfoQuest FP software (version 4.50, Bio-Rad Laboratories, Inc).

**
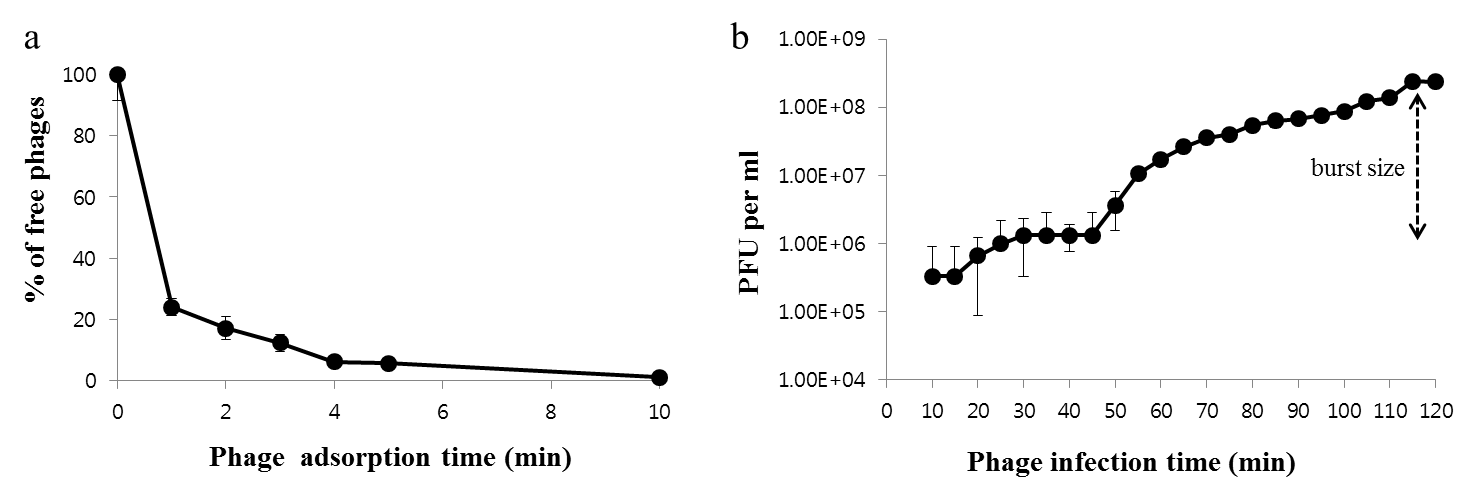
**

Figure S2. The adsorption rate (A) and one-step growth curve (B) of *A. baumannii* phage Bϕ-R2096 on *A. baumannii* strain YMC13/03/R2096. Data on phage adsorption showed that 83% of the phage was adsorbed on the host bacteria after 2 min. Mean ± standard deviation data are from triplicate experiments (A). The results show that the latent period was approximately 50 min, and the burst size was about 142 PFU/ml after 115 min (B).


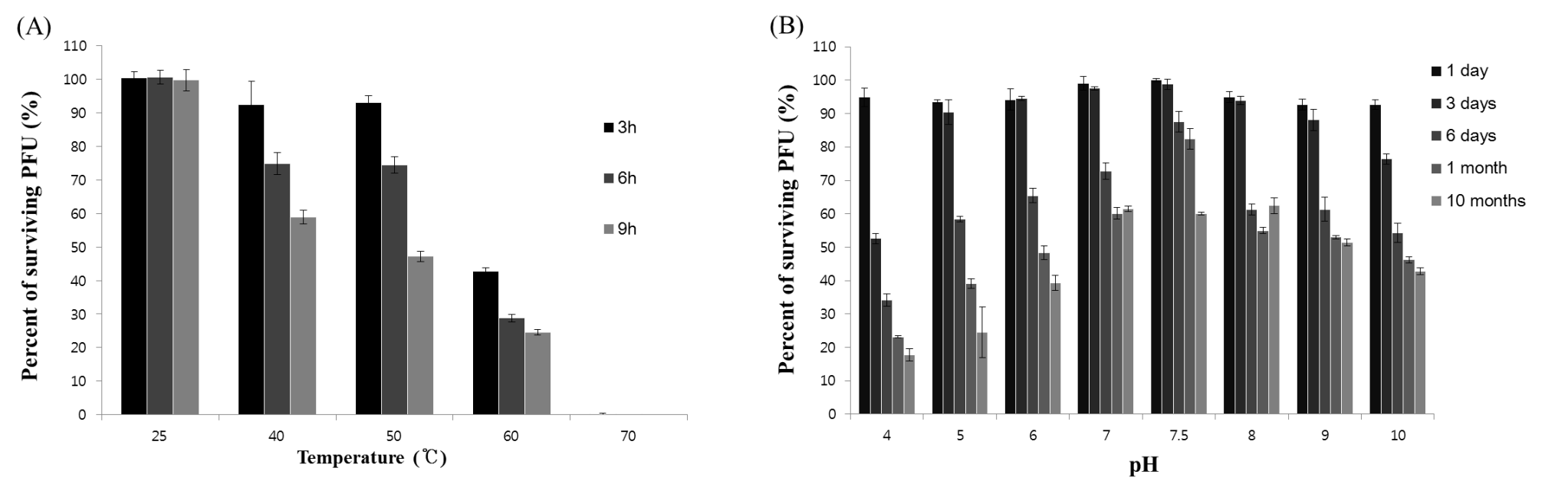


Figure S3. Temperature and pH stability of *A. baumannii* phage Βφ-R2096 on *A. baumannii* strain YMC13/03/R2096. Phages were incubated for 3, 6, and 9 h at 25°C, 40°C, 50°C, 60°C, and 70°C, and stability was calculated as the percentage of surviving phages (A). Phages were incubated at pH 4–10 for 1, 3, and 6 days, 1 month, and 10 months at 4°C, and stability was calculated as the percentage of surviving phages (B). The results shown are mean ± standard deviation.


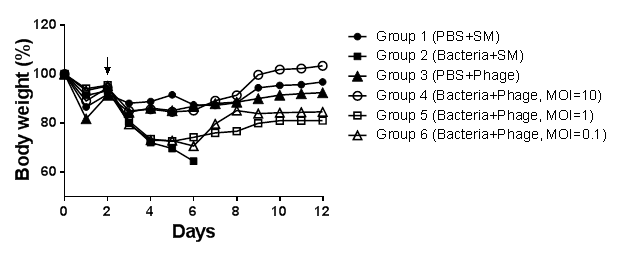


Figure S4. Body weight of C57BL/6 mice infected with *A. baumannii* phage Bϕ-R2096, *A. baumannii* YMC13/03/R2096, or both. Group 1 (control): PBS+SM buffer treatment; Group 2: bacteria-only-treatment; Group 3: phage-only-treatment; Groups 4–6: postinfection phage treatment (MOI 0.1, 1, 10) 30 min after bacterial infection. Body weights were measured daily. The black arrow indicates a second cyclophosphamide (CP) administration and the first bacterial infection. Values given are averages (n = 6 mice)


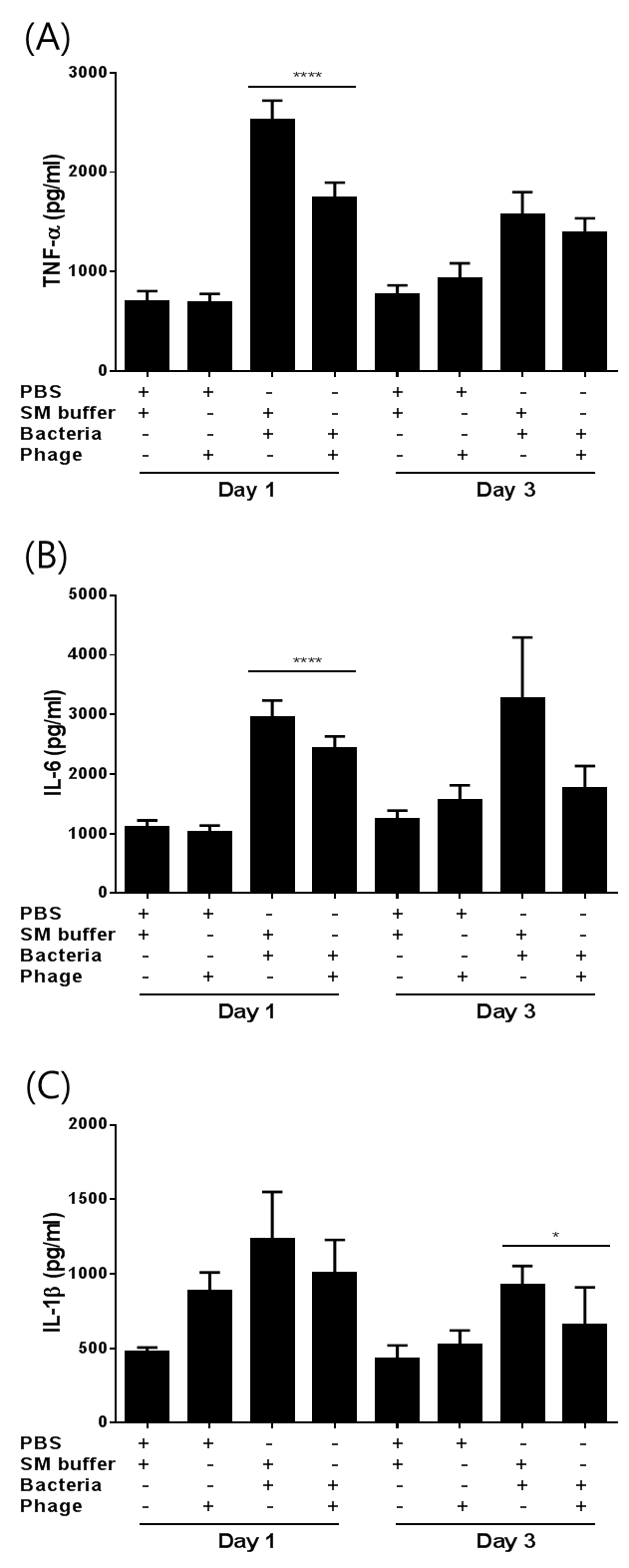


Figure S5. The concentration of cytokines (TNF-α, IL-6, and IL-1β) in the lungs of mice on days 1 and 3 after treatment with *A. baumannii* YMC13/03/R2096, phage Bϕ-R2096, or both. The one-way ANOVA with Tukey`s multiple comparisons test was used to compare the cytokine levels. Significant differences (****p<0.0001) were observed on each day.

Table S1. Antibiotic resistance profiles of carbapenem-resistant *A. baumannii* clinical strains used in this study^a^

| Host stain | MLST^b^ | OTC^c^ | MHT^d^ | Amikacin | Ampicillin/ Sulbactam | Ceftazidime | Colistin | Cefepime | Cefotaxime | Gentamicin | Imipenem | Levofloxacin | Meropenem | Minocycline | Piperacillin/ Tazobactam | Cotrimoxazole | Tigecycline |
| --- | --- | --- | --- | --- | --- | --- | --- | --- | --- | --- | --- | --- | --- | --- | --- | --- | --- |
| YMC13/03/R2096 | 357 | OXA-66-like | + | ≥64 (R) | ≤16 (I) | ≥64 (R) | ≤0.5 (S) | ≥64 (R) | ≥64 (R) | ≥16 (R) | ≥16 (R) | ≥8 (R) | ≥16 (R) | ≤8 (I) | ≥128 (R) | ≥320 (R) | ≤2 (S) |
| YMC13/01/R1400 | 357 | OXA-66-like | + | ≥64 (R) | ≥32 (R) | ≥64 (R) | ≤0.5 (S) | ≥64 (R) | ≥64 (R) | ≥16 (R) | ≥16 (R) | ≥8 (R) | ≥16 (R) | ≤8 (I) | ≥128 (R) | ≥320 (R) | ≤1 (S) |
| YMC13/01/R1224 | 357 | OXA-66-like | + | ≥64 (R) | ≥32 (R) | ≥64 (R) | ≤0.5 (S) | ≥64 (R) | ≥64 (R) | ≥16 (R) | ≥16 (R) | ≥8 (R) | ≥16 (R) | ≤4 (S) | ≥128 (R) | ≥320 (R) | ≤1 (S) |
| YMC13/01/R1919 | 357 | OXA-66-like | + | ≥64 (R) | ≥32 (R) | ≥64 (R) | ≤0.5 (S) | ≥64 (R) | ≥64 (R) | ≥16 (R) | ≥16 (R) | ≥8 (R) | ≥16 (R) | ≤4 (S) | ≥128 (R) | ≥320 (R) | ≤1 (S) |
| YMC13/01/R187 | 357 | OXA-66-like | + | ≤8 (S) | ≥32 (R) | ≥64 (R) | ≤0.5 (S) | ≥64 (R) | ≥64 (R) | ≤2 (S) | ≥16 (R) | ≥8 (R) | ≥16 (R) | ≤8 (I) | ≥128 (R) | ≥320 (R) | ≤2 (S) |
| YMC13/01/R2058 | 357 | OXA-66-like | + | ≥64 (R) | ≤8 (S) | ≥64 (R) | ≤0.5 (S) | ≥64 (R) | ≥64 (R) | ≥16 (R) | ≥16 (R) | ≤4 (I) | ≥16 (R) | ≤4 (S) | ≥128 (R) | ≥320 (R) | ≤1(S) |
| YMC13/02/R1138 | 357 | OXA-66-like | + | ≥64 (R) | ≤16 (I) | ≥64 (R) | ≤0.5 (S) | ≥64 (R) | ≥64 (R) | ≥16 (R) | ≥16 (R) | ≥8 (R) | ≥16 (R) | ≤8 (I) | ≥128 (R) | ≥320 (R) | ≤2 (S) |
| YMC13/01/R249 | 357 | OXA-66-like | + | ≤8 (S) | ≥32 (R) | ≥64 (R) | ≤0.5 (S) | ≥64 (R) | ≥64 (R) | ≤4 (S) | ≥16 (R) | ≥8 (R) | ≥16 (R) | ≤8 (I) | ≥128 (R) | ≥320 (R) | ≤1(S) |
| YMC13/01/R280 | 357 | OXA-66-like | + | ≤8 (S) | ≥32 (R) | ≥64 (R) | ≤0.5 (S) | ≥64 (R) | ≥64 (R) | ≤1 (S) | ≥16 (R) | ≥8 (R) | ≥16 (R) | ≤4 (S) | ≥128 (R) | ≥320 (R) | ≤2 (S) |
| YMC13/01/R224 | 357 | OXA-66-like | + | ≥64 (R) | ≤8 (S) | ≥64 (R) | ≤0.5 (S) | ≥64 (R) | ≥64 (R) | ≥16 (R) | ≥16 (R) | ≤4 (I) | ≥16 (R) | ≤8 (I) | ≥128 (R) | ≥320 (R) | ≤1(S) |
| YMC13/01/R656 | 357 | OXA-66-like | + | ≥64 (R) | ≥32 (R) | ≥64 (R) | ≤0.5 (S) | ≥64 (R) | ≥64 (R) | ≥16 (R) | ≥16 (R) | ≥8 (R) | ≥16 (R) | ≤4 (S) | ≥128 (R) | ≥320 (R) | ≤2 (S) |
| YMC13/03/R12096 | 357 | OXA-66-like | + | ≥64 (R) | ≥32 (R) | ≥64 (R) | ≤0.5 (S) | ≥64 (R) | ≥64 (R) | ≥16 (R) | ≥16 (R) | ≥8 (R) | ≥16 (R) | ≤8 (I) | ≥128 (R) | ≥320 (R) | ≤1 (S) |
| YMC13/01/R317 | 357 | OXA-66-like | + | ≥64 (R) | ≥32 (R) | ≥64 (R) | ≤0.5 (S) | ≥64 (R) | ≥64 (R) | ≥16 (R) | ≥16 (R) | ≥8 (R) | ≥16 (R) | ≤8 (I) | ≥128 (R) | ≥320 (R) | ≤2 (S) |
| YMC13/01/R129 | 357 | OXA-66-like | + | ≥64 (R) | ≤8 (S) | ≥64 (R) | ≤0.5 (S) | ≥64 (R) | ≥64 (R) | ≥16 (R) | ≥16 (R) | ≥8 (R) | ≥16 (R) | ≤8 (I) | ≥128 (R) | ≥320 (R) | ≤2 (S) |
| YMC13/01/R3197 | 357 | OXA-66-like | + | ≥64 (R) | ≥32 (R) | ≥64 (R) | ≤0.5 (S) | ≥64 (R) | ≥64 (R) | ≥16 (R) | ≥16 (R) | ≥8 (R) | ≥16 (R) | ≤4 (S) | ≥128 (R) | ≥320 (R) | ≤1(S) |
| YMC13/03/R720 | 357 | OXA-66-like | + | ≥64 (R) | ≤8 (S) | ≥64 (R) | ≤0.5 (S) | ≥64 (R) | ≥64 (R) | ≥16 (R) | ≥16 (R) | ≥8 (R) | ≥16 (R) | ≤8 (I) | ≥128 (R) | ≥320 (R) | ≤0.5 (S) |
| YMC13/02/R291 | 357 | OXA-66-like | + | ≥64 (R) | ≤8 (S) | ≥64 (R) | ≤0.5 (S) | ≥64 (R) | ≥64 (R) | ≥16 (R) | ≥16 (R) | ≥8 (R) | ≥16 (R) | ≤4 (S) | ≥128 (R) | ≥320 (R) | ≤2 (S) |
| YMC13/02/R319 | 357 | OXA-66-like | + | ≥64 (R) | ≤8 (S) | ≥64 (R) | ≤0.5 (S) | ≥64 (R) | ≥64 (R) | ≥16 (R) | ≥16 (R) | ≥8 (R) | ≥16 (R) | ≤8 (I) | ≥128 (R) | ≥320 (R) | ≤2 (S) |
| YMC13/02/R401 | 357 | OXA-66-like | + | ≥64 (R) | ≤8 (S) | ≥64 (R) | ≤0.5 (S) | ≥64 (R) | ≥64 (R) | ≥16 (R) | ≥16 (R) | ≥8 (R) | ≥16 (R) | ≤1 (I) | ≥128 (R) | ≥320 (R) | ≤1 (S) |
| YMC13/02/R427 | 357 | OXA-66-like | + | ≥64 (R) | ≤8 (S) | ≥64 (R) | ≤0.5 (S) | ≥64 (R) | ≥64 (R) | ≥16 (R) | ≥16 (R) | ≥8 (R) | ≥16 (R) | ≤8 (I) | ≥128 (R) | ≥320 (R) | ≤2 (S) |

^a^ Antibiotic resistance was determined by the disk diffusion test method. ‘Susceptible’, ‘Intermediate’ and ‘Resistant’ are abbreviated as ‘S’, ‘I’ and ‘R’.

^b^MLST: multilocus sequence typing, ^c^ OTC: OXA-type carbapenemase,^d^ MHT: modified Hodge test.

Table S2. *A. baumannii* phage Bϕ-R2096 ORFs summary

| **Gene no.** | **Range** | | **Initiation  codon** | **Strand** | **Length  (bp)** | **Putative function** | **Annotation source** | **E-value** | **NCBI blastP identity (%)** | **NCBI-Bank**  **accession number** |
| --- | --- | --- | --- | --- | --- | --- | --- | --- | --- | --- |
|  | **start** | **stop** |  |  |  |  |  |  |  |  |
| ORF 1 | 46 | 744 | ATG | - | 699 |  |  |  |  |  |
| ORF 2 | 737 | 1180 | ATG | - | 444 |  |  |  |  |  |
| ORF 3 | 1177 | 1389 | ATG | - | 213 |  |  |  |  |  |
| ORF 4 | 1466 | 1882 | ATG | - | 417 |  |  |  |  |  |
| ORF 5 | 1893 | 2060 | GTG | - | 168 |  |  |  |  |  |
| ORF 6 | 2138 | 2374 | ATG | - | 237 |  |  |  |  |  |
| ORF 7 | 2398 | 2607 | ATG | - | 210 |  |  |  |  |  |
| ORF 8 | 2653 | 3030 | ATG | - | 378 |  |  |  |  |  |
| ORF 9 | 3113 | 3229 | ATG | - | 117 |  |  |  |  |  |
| ORF 10 | 3231 | 3461 | ATG | - | 231 |  |  |  |  |  |
| ORF 11 | 3479 | 3775 | ATG | - | 297 |  |  |  |  |  |
| ORF 12 | 3821 | 4168 | GTG | - | 348 |  |  |  |  |  |
| ORF 13 | 4161 | 4451 | ATG | - | 291 |  |  |  |  |  |
| ORF 14 | 4454 | 4726 | ATG | - | 273 |  |  |  |  |  |
| ORF 15 | 4742 | 5176 | ATG | - | 435 |  |  |  |  |  |
| ORF 16 | 5151 | 5342 | ATG | - | 192 |  |  |  |  |  |
| ORF 17 | 5342 | 5554 | ATG | - | 213 |  |  |  |  |  |
| ORF 18 | 5541 | 5693 | ATG | - | 153 |  |  |  |  |  |
| ORF 19 | 5690 | 6178 | ATG | - | 489 |  |  |  |  |  |
| ORF 20 | 6192 | 6554 | ATG | - | 363 |  |  |  |  |  |
| ORF 21 | 6583 | 7809 | ATG | - | 1227 |  |  |  |  |  |
| ORF 22 | 7810 | 8022 | ATG | - | 213 |  |  |  |  |  |
| ORF 23 | 8022 | 8261 | ATG | - | 240 |  |  |  |  |  |
| ORF 24 | 8254 | 8517 | ATG | - | 264 |  |  |  |  |  |
| ORF 25 | 8734 | 9114 | ATG | - | 381 |  |  |  |  |  |
| ORF 26 | 9196 | 9360 | ATG | - | 165 |  |  |  |  |  |
| ORF 27 | 9364 | 9564 | ATG | - | 201 |  |  |  |  |  |
| ORF 28 | 9557 | 9871 | ATG | - | 315 |  |  |  |  |  |
| ORF 29 | 9852 | 10142 | ATG | - | 291 |  |  |  |  |  |
| ORF 30 | 10139 | 10390 | ATG | - | 252 |  |  |  |  |  |
| ORF 31 | 10401 | 10607 | ATG | - | 207 |  |  |  |  |  |
| ORF 32 | 11077 | 11376 | ATG | + | 300 |  |  |  |  |  |
| ORF 33 | 11396 | 12028 | ATG | + | 633 |  |  |  |  |  |
| ORF 34 | 12032 | 14794 | ATG | + | 2763 | Putative tail fiber | [*Acinetobacter* phage AM24] | 3e-133 | 71 | APD20249.1 |
| ORF 35 | 14921 | 15307 | TTG | + | 387 | Putative endolysin | [Acinetobacter phage vB_AbaM_B9] | 6e-16 | 31 | YP_007004738.1 |
| ORF 36 | 15332 | 15847 | ATG | + | 516 |  |  |  |  |  |
| ORF 37 | 18958 | 19239 | ATG | + | 282 |  |  |  |  |  |
| ORF 38 | 19879 | 20193 | GTG | + | 315 |  |  |  |  |  |
| ORF 39 | 20409 | 20903 | ATG | + | 495 | Putative HNH endonuclease | [*Staphylococcus* phage 6ec] | 4e-23 | 48 | YP_009042633.1 |
| ORF 40 | 21742 | 22905 | ATG | + | 1164 | Putative terminase  large subunit | [*Acinetobacter* phage AM24] | 0 | 99 | YP_007002481.1 |
| ORF 41 | 23056 | 24417 | ATG | + | 1362 | Putative portal protein | [Acinetobacter phage vB_AbaM_B9] | 2e-96 | 36 | AHY26734.1 |
| ORF 42 | 24425 | 25999 | ATG | + | 1575 | Putative capsid protein | [*Vibrio* phage PWH3a-P1] | 3e-32 | 31 | YP_007676023.1 |
| ORF 43 | 26018 | 26482 | ATG | + | 465 | Putative structural protein | [*Acinetobacter* phage vB_AbaM_Acibel004] | 1e-20 | 36 | AHY26736.1 |
| ORF 44 | 26588 | 27658 | ATG | + | 1071 | Putative major capsid protein | [*Acinetobacter* phage vB_AbaM_Acibel004] | 2e-59 | 32 | AHY26737.1 |
| ORF 45 | 27776 | 28324 | ATG | + | 549 |  |  |  |  |  |
| ORF 46 | 28334 | 28939 | ATG | + | 606 | Putative tail protein | [*Acinetobacter* phage vB_AbaM_Acibel004] | 0.016 | 24 | AHY26742.1 |
| ORF 47 | 28958 | 29368 | ATG | + | 411 |  |  |  |  |  |
| ORF 48 | 29371 | 29937 | ATG | + | 567 |  |  |  |  |  |
| ORF 49 | 29951 | 30979 | ATG | + | 1029 | Putative structural protein | [*Pseudomonas* phage PaP1] | 3e-28 | 32 | YP_007236467.1 |
| ORF 50 | 31017 | 31544 | ATG | + | 528 | Putative structural protein | [*Pseudomonas* phage KPP10] | 2e-35 | 38 | YP_004306761.1 |
| ORF 51 | 31601 | 32167 | ATG | + | 567 | Putative structural protein | [*Acinetobacter* phage vB_AbaM_Acibel004] | 5.00E-14 | 28 | AHY26747.1 |
| ORF 52 | 32180 | 32785 | ATG | + | 606 |  |  |  |  |  |
| ORF 53 | 32811 | 33236 | ATG | + | 426 |  |  |  |  |  |
| ORF 54 | 33278 | 33445 | ATG | + | 168 |  |  |  |  |  |
| ORF 55 | 33461 | 35584 | ATG | + | 2124 | Putative tape measure protein | [*Acinetobacter* phage vB_AbaM_Acibel004] | 1e-11 | 23 | AHY26751.1 |
| ORF 56 | 35622 | 36515 | ATG | + | 894 | Putative structural protein | [*Acinetobacter* phage vB_AbaM_Acibel004] | 0.015 | 24 | AHY26752.1 |
| ORF 57 | 36530 | 36877 | ATG | + | 348 |  |  |  |  |  |
| ORF 58 | 36890 | 38086 | ATG | + | 1197 |  |  |  |  |  |
| ORF 59 | 38097 | 38840 | ATG | + | 744 | Putative baseplate protein | [*Pseudomonas* phage KPP10] | 4e-35 | 38 | YP_004306770.1 |
| ORF 60 | 38867 | 39277 | ATG | + | 411 |  |  |  |  |  |
| ORF 61 | 39287 | 40750 | ATG | + | 1464 |  |  |  |  |  |
| ORF 62 | 40759 | 41385 | ATG | + | 627 | Putative tail component | [*Acinetobacter* phage vB_AbaM_Acibel004] | 3e-20 | 40 | AHY26758.1 |
| ORF 63 | 41387 | 42466 | ATG | + | 1080 | Putative tail fiber protein | [*Pseudomonas* phage PAK_P5] | 3e-12 | 44 | YP_008856902.1 |
| ORF 64 | 42479 | 42865 | GTG | + | 387 |  |  |  |  |  |
| ORF 65 | 42887 | 43213 | ATG | + | 327 |  |  |  |  |  |
| ORF 66 | 43215 | 44459 | ATG | + | 1245 | Putative tail fiber protein | [Acinetobacter phage AM24] | 0.0 | 55 | YP_009005094.1 |
| ORF 67 | 44461 | 45027 | ATG | + | 567 |  |  |  |  |  |
| ORF 68 | 45054 | 45728 | ATG | + | 675 | Putative endolysin/autolysin | [Acinetobacter phage AM24] | 1e-160 | 56 | YP_006383807.1 |
| ORF 69 | 45750 | 46031 | ATG | + | 282 |  |  |  |  |  |
| ORF 70 | 46107 | 48878 | GTG | - | 2772 | Putative ribonucleoside-diphosphatereductase  alpha chain | [Acinetobacter phage AM24] | 0.0 | 63 | YP_007236524.1 |
| ORF 71 | 48889 | 49317 | ATG | - | 429 | Putative ribonucleoside-diphosphatereductase  alpha chain | [Acinetobacter phage AM24] | 4e-99 | 40 | YP_007236523.1 |
| ORF 72 | 49542 | 50090 | ATG | - | 549 | Putative HNH endonuclease | [*Erwinia* phage phiEa100] | 1e-07 | 33 | YP_007237524.1 |
| ORF 73 | 50249 | 51016 | ATG | - | 768 | Putative ribonucleotide-diphosphatereductase  beta subunit | [*Pseudomonas* phage PAK_P1] | 1e-75 | 61 | YP_004327264.1 |
| ORF 74 | 51096 | 51572 | ATG | - | 477 |  |  |  |  |  |
| ORF 75 | 52177 | 52911 | ATG | + | 735 |  |  |  |  |  |
| ORF 76 | 52944 | 53567 | ATG | + | 624 |  |  |  |  |  |
| ORF 77 | 53659 | 54150 | ATG | + | 492 |  |  |  |  |  |
| ORF 78 | 54158 | 54427 | ATG | + | 270 |  |  |  |  |  |
| ORF 79 | 54510 | 54797 | ATG | + | 288 |  |  |  |  |  |
| ORF 80 | 54811 | 56847 | GTG | + | 2037 | Putative DNA primase/helicase | [*Pseudomonas* phage vB_PaeM_C2-10_Ab1] | 3e-129 | 37 | YP_007236917.1 |
| ORF 81 | 56888 | 59392 | TTG | + | 2505 | Putative DNA polymerase | [*Acinetobacter* phage AM24] | 0 | 93 | APD20293.1 |
| ORF 82 | 59464 | 60723 | ATG | + | 1260 | Putative ATPase | [*Salmonella* phage 7-11] | 7e-46 | 43 | YP_004782500.1 |
| ORF 83 | 60766 | 61002 | ATG | + | 237 |  |  |  |  |  |
| ORF 84 | 60989 | 61228 | ATG | + | 240 |  |  |  |  |  |
| ORF 85 | 61485 | 62144 | ATG | + | 660 |  |  |  |  |  |
| ORF 86 | 62159 | 62704 | ATG | + | 546 |  |  |  |  |  |
| ORF 87 | 62745 | 63980 | ATG | + | 1236 | Putative exodeoxyribonuclease | [*Acinetobacter* phage vB_AbaM_Acibel004] | 4e-36 | 28 | AHY26626.1 |
| ORF 88 | 64062 | 64736 | ATG | + | 675 | Putative HNH endonuclease protein | [*Acinetobacter* phage vB_AbaM_Acibel004] | 5e-32 | 38 | AHY26628.1 |
| ORF 89 | 64750 | 65727 | ATG | + | 978 | Putative DNA polymerase II | [*Acinetobacter* phage vB_AbaM_Acibel004] | 2e-86 | 44 | AHY26629.1 |
| ORF 90 | 65742 | 66653 | ATG | + | 912 |  |  |  |  |  |
| ORF 91 | 66619 | 66756 | ATG | + | 138 |  |  |  |  |  |
| ORF 92 | 66743 | 67771 | TTG | + | 1029 | Putative thymidylate synthase | [*Pseudomonas* phage vB_PaeM_C2-10_Ab1] | 7e-94 | 48 | YP_004327262.1 |
| ORF 93 | 67782 | 68171 | ATG | + | 390 |  |  |  |  |  |
| ORF 94 | 68261 | 70030 | ATG | + | 1770 | Putative phosphate starvation-inducible protein | [*Celeribacter* phage P12053L] | 1e-32 | 41 | YP_006560919.1 |
| ORF 95 | 70030 | 70419 | ATG | + | 390 |  |  |  |  |  |
| ORF 96 | 70496 | 70681 | ATG | + | 186 |  |  |  |  |  |
| ORF 97 | 70684 | 71109 | ATG | + | 426 |  |  |  |  |  |
| ORF 98 | 71193 | 71765 | ATG | + | 573 |  |  |  |  |  |
| ORF 99 | 71845 | 72237 | ATG | + | 393 |  |  |  |  |  |
| ORF 100 | 72300 | 72602 | ATG | + | 303 |  |  |  |  |  |
| ORF 101 | 72602 | 73117 | ATG | + | 516 |  |  |  |  |  |
| ORF 102 | 73148 | 73354 | ATG | + | 207 |  |  |  |  |  |
| ORF 103 | 73358 | 73621 | ATG | + | 264 |  |  |  |  |  |
| ORF 104 | 73666 | 74493 | ATG | + | 828 |  |  |  |  |  |
| ORF 105 | 74540 | 74707 | ATG | + | 168 |  |  |  |  |  |
| ORF 106 | 74722 | 75072 | ATG | + | 351 |  |  |  |  |  |
| ORF 107 | 75113 | 75319 | ATG | + | 207 |  |  |  |  |  |
| ORF 108 | 75316 | 75528 | ATG | + | 213 |  |  |  |  |  |
| ORF 109 | 75615 | 76046 | ATG | + | 432 |  |  |  |  |  |
| ORF 110 | 76048 | 76182 | ATG | + | 135 |  |  |  |  |  |
| ORF 111 | 76198 | 76764 | GTG | + | 567 |  |  |  |  |  |
| ORF 112 | 76761 | 77147 | ATG | + | 387 |  |  |  |  |  |
| ORF 113 | 77224 | 77595 | GTG | + | 372 |  |  |  |  |  |
| ORF 114 | 77601 | 77924 | TTG | + | 324 |  |  |  |  |  |
| ORF 115 | 78019 | 78459 | ATG | + | 441 |  |  |  |  |  |
| ORF 116 | 78609 | 78743 | ATG | + | 135 |  |  |  |  |  |
| ORF 117 | 78721 | 78864 | ATG | + | 144 |  |  |  |  |  |
| ORF 118 | 78861 | 79085 | ATG | + | 225 |  |  |  |  |  |
| ORF 119 | 79075 | 79248 | ATG | + | 174 |  |  |  |  |  |
| ORF 120 | 79250 | 79456 | ATG | + | 207 |  |  |  |  |  |
| ORF 121 | 79466 | 79852 | ATG | + | 387 |  |  |  |  |  |
| ORF 122 | 79862 | 80077 | ATG | + | 216 |  |  |  |  |  |
| ORF 123 | 80077 | 80289 | ATG | + | 213 |  |  |  |  |  |
| ORF 124 | 80298 | 80519 | ATG | + | 222 |  |  |  |  |  |
| ORF 125 | 80516 | 80743 | GTG | + | 228 |  |  |  |  |  |
| ORF 126 | 80772 | 80960 | ATG | + | 189 |  |  |  |  |  |
| ORF 127 | 80963 | 81157 | ATG | + | 195 |  |  |  |  |  |
| ORF 128 | 81135 | 81284 | ATG | + | 150 |  |  |  |  |  |
| ORF 129 | 81294 | 82487 | ATG | + | 1194 | Putative AAA family ATPase | [*Staphylococcus* phage JD007] | 1e-31 | 32 | YP_007112820.1 |
| ORF 130 | 82617 | 83510 | ATG | + | 894 |  |  |  |  |  |
| ORF 131 | 83513 | 83791 | ATG | + | 279 |  |  |  |  |  |
| ORF 132 | 83791 | 84084 | ATG | + | 294 |  |  |  |  |  |
| ORF 133 | 84115 | 84387 | ATG | + | 273 |  |  |  |  |  |
| ORF 134 | 84391 | 84852 | GTG | + | 462 |  |  |  |  |  |
| ORF 135 | 84937 | 85401 | ATG | + | 465 |  |  |  |  |  |
| ORF 136 | 85394 | 85642 | ATG | + | 249 |  |  |  |  |  |
| ORF 137 | 85642 | 85959 | ATG | + | 318 |  |  |  |  |  |
| ORF 138 | 86145 | 86414 | ATG | + | 270 |  |  |  |  |  |
| ORF 139 | 86411 | 86668 | ATG | + | 258 |  |  |  |  |  |
| ORF 140 | 86671 | 87021 | ATG | + | 351 |  |  |  |  |  |
| ORF 141 | 87120 | 87440 | ATG | + | 321 |  |  |  |  |  |
| ORF 142 | 88195 | 88320 | GTG | + | 126 |  |  |  |  |  |
| ORF 143 | 88482 | 89042 | ATG | + | 561 |  |  |  |  |  |
| ORF 144 | 89166 | 89435 | ATG | + | 270 |  |  |  |  |  |
| ORF 145 | 89974 | 90117 | ATG | + | 144 |  |  |  |  |  |
| ORF 146 | 90199 | 90729 | ATG | + | 531 |  |  |  |  |  |
| ORF 147 | 90776 | 90940 | ATG | + | 165 |  |  |  |  |  |
| ORF 148 | 91045 | 91188 | ATG | + | 144 |  |  |  |  |  |
| ORF 149 | 91740 | 91946 | ATG | + | 207 |  |  |  |  |  |
| ORF 150 | 91961 | 92143 | ATG | + | 183 |  |  |  |  |  |
| ORF 151 | 92430 | 92639 | ATG | + | 210 |  |  |  |  |  |
| ORF 152 | 92776 | 93105 | ATG | + | 330 |  |  |  |  |  |
| ORF 153 | 93342 | 93698 | ATG | + | 357 |  |  |  |  |  |
| ORF 154 | 93743 | 93862 | TTG | + | 120 |  |  |  |  |  |
| ORF 155 | 94455 | 94691 | ATG | - | 237 |  |  |  |  |  |
| ORF 156 | 94681 | 94932 | ATG | - | 252 |  |  |  |  |  |
| ORF 157 | 94925 | 95167 | ATG | - | 243 |  |  |  |  |  |
| ORF 158 | 95161 | 95730 | GTG | - | 570 |  |  |  |  |  |
| ORF 159 | 95752 | 96309 | ATG | - | 558 |  |  |  |  |  |
| ORF 160 | 96325 | 96693 | ATG | - | 369 |  |  |  |  |  |
| ORF 161 | 96745 | 97002 | ATG | - | 258 |  |  |  |  |  |
| ORF 162 | 96999 | 98138 | GTG | - | 1140 |  |  |  |  |  |

**Supplementary References**

1. Karumidze N, Kusradze I, Rigvava S, Goderdzishvili M, Rajakumar K, Alavidze Z. Isolation and characterisation of lytic bacteriophages of *Klebsiella pneumoniae* and *Klebsiella oxytoca*. Curr Microbiol. 2013;66(3):251-8.

2. Lin NT, Chiou PY, Chang KC, Chen LK, Lai MJ. Isolation and characterization of phi AB2: a novel bacteriophage of *Acinetobacter baumannii*. Res Microbiol. 2010;161(4):308-14.

3. Yang H, Liang L, Lin S, Jia S. Isolation and characterization of a virulent bacteriophage AB1 of *Acinetobacter baumannii*. BMC Microbiol. 2010;10:131.
